# Risk factors on surgical compliance and its impact on survival outcomes in meningioma patients: a SEER-based retrospective propensity-score matched analysis

**DOI:** 10.1186/s12893-024-02326-1

**Published:** 2024-01-30

**Authors:** Shengkai Yang, Hongwei Teng, Yingdan Wang, Kangkang Ji, Weihua Chen, Hai Zhou

**Affiliations:** 1 Department of Neurosurgery, Binhai County People’s Hospital, Yancheng, China; 2 Department of Pediatric, Binhai County People’s Hospital, Yancheng, China; 3 Department of Central Laboratory, Binhai County People’s Hospital, Yancheng, China

**Keywords:** Meningioma, Surgical compliance, Overall survival, Nomogram, SEER, PSM

## Abstract

**Background:**

This study aimed to analyze the effect of surgical compliance on the survival outcome of patients with meningioma and explore the factors affecting surgical compliance.

**Methods:**

We selected data from the Surveillance, Epidemiology, and End Results database for 122,632 meningioma patients diagnosed between 2004 and 2018. The effect of surgical compliance on patients’ overall survival (OS) was analyzed through Cox regression and Kaplan–Meier curves. Independent risk factors for surgical compliance were identified through multifactorial logistic regression analyses to construct diagnostic nomograms, further assessed by receiver operating characteristic curves. Furthermore, we used univariate and multivariate logistic regression analyses to evaluate relevant variables linked to adherence with meningioma surgery. Moreover, 1:1 propensity score matching was applied to assess the validity of the results in patients with favorable and poor surgical compliance.

**Results:**

A total of 48,735 were eligible from the initial cohort of 122,632 patients with meningioma. Among them, 45,038 (92.40%) exhibited good surgical compliance, while 3697 (7.60%) had poor surgical compliance. The rate of patients with good surgical compliance was significantly higher than that of patients with inadequate surgical compliance (*p* < 0.001). Moreover, surgical compliance is an independent prognostic factor for OS in meningioma patients. Univariate Cox regression analysis indicated that individuals with poor surgical compliance demonstrated lower OS rates than those with good surgical compliance (hazard ratio [HR 2.404; 95% confidence interval [CI] 2.276–2.54, *p* < 0.001], consistent with the observation in the multivariate analysis (HR 1.564; 95% CI 1.471–1.663, *p* < 0.001). We developed a prediction model using seven variables: age, sex, race, tumor behavior recode, tumor size, family income, and residential setting (*p* < 0.05). Surgical compliance was associated with patient age, sex, race, tumor behavior recode, tumor size, family income, and residential setting by logistic regression analysis.

**Conclusions:**

Surgical compliance emerged as an independent prognostic factor for survival in patients with meningioma. Poor surgical compliance was associated with older age, black and other races, females, advanced-stage tumors, larger tumor size, lower household income, and rural residence. When patients experienced these conditions, OS was shorter, requiring more aggressive treatment.

## Introduction

Meningioma accounts for 38.3% of all types of tumors in the central nervous system (CNS), and it is the most common benign tumor of the CNS in the United States [1]. Among primary meningioma, 97% are benign meningioma, and only 3% are malignant meningioma. Women are reportedly more susceptible to CNS meningioma than men [2, 3]. Besides primarily originating from arachnoid granule cap cells, these tumors could also arise from the meninges, homologous mesenchymal cells, scattered meningeal cells, astrocytes, and embryonic neuroectodermal cells [4]. Meningioma occur primarily in the cranial cavity of the body [5], with only about 10% being extracranial spinal cord lesions [6].

Surgery represents the standard treatment approach for meningioma, and most patients achieve a cure through surgical removal alone. Neurosurgeons recommend surgical removal of intracranial tumors to alleviate symptoms secondary to edema around the meningioma, significant nerve compression, headache due to tumor occupancy, and even mental retardation. The main goal of the surgery is to remove the meningioma, including the dura mater, skull, and compressed vascular nerves invaded by the tumor. The standard grading method for the extent of meningioma resection is the Simpson classification, which divides resection into five grades and provides an excellent assessment of the prognosis [7–9]. A more thorough tumor removal reduces the risk of recurrence, increases the likelihood of a cure, and improves the prognosis for patients [10–19]. Surgical resection offers better clinical benefits than conservative treatment. However, some patients with meningioma recommended for surgery do not undergo the procedure for multiple factors, which could be affected by clinical pathologic characteristics, patient fears, misinformation, logistical challenges and other elusive reasons. For benign, borderline, and malignant meningiomas, surgical resection improved survival [1, 12]. Surgical intervention has the potential to reduce neurologic symptoms and achieve favorable, long-term outcomes [13]. Thus, surgical compliance might significantly influence the prognosis of meningioma. Therefore, it is important to analyze the effects of surgical noncompliance on survival prognosis in meningioma patients and identify the relevant factors affecting adherence to improve patient outcomes.

The decision for surgery requires a detailed assessment of the severity of the patient’s symptoms, the rate of disease progression, the location of the meningioma, the likelihood of successful tumor resection, the extent of resection, and the potential benefits of the surgery. After thorough communication with the patients and their families, the doctor should make the final treatment decision [20]. Surgical noncompliance is a severe treatment deficit influenced by various factors, and we hypothesized that demographic and clinical pathologic characteristics influence it. Based on this hypothesis, we aimed to identify the relevant variables contributing to surgical noncompliance in this retrospective analysis.

## Materials and methods

### Ethics

This study used publicly available SEER cancer registry data. Because patient data in the SEER database were de-identified, signed informed consent was waived for the present study.

### Data sources

The Surveillance Epidemiology and End Results (SEER) database, a public database commonly used in clinical practice, was used for this study. This database was particularly suited for this study because its comprehensive coverage of tumor types and clinical information, such as sex, age, surgical compliance, and tumor TNM stage, aligned well with the study’s objectives. The SEER database was used to identify patients diagnosed with meningioma between 2004 and 2018 from selected states and counties, including about a third of the US population [21]. This study utilized custom data with additional treatment fields from 18 centers in SEER database version 8.3.5.

**Fig. 1:**
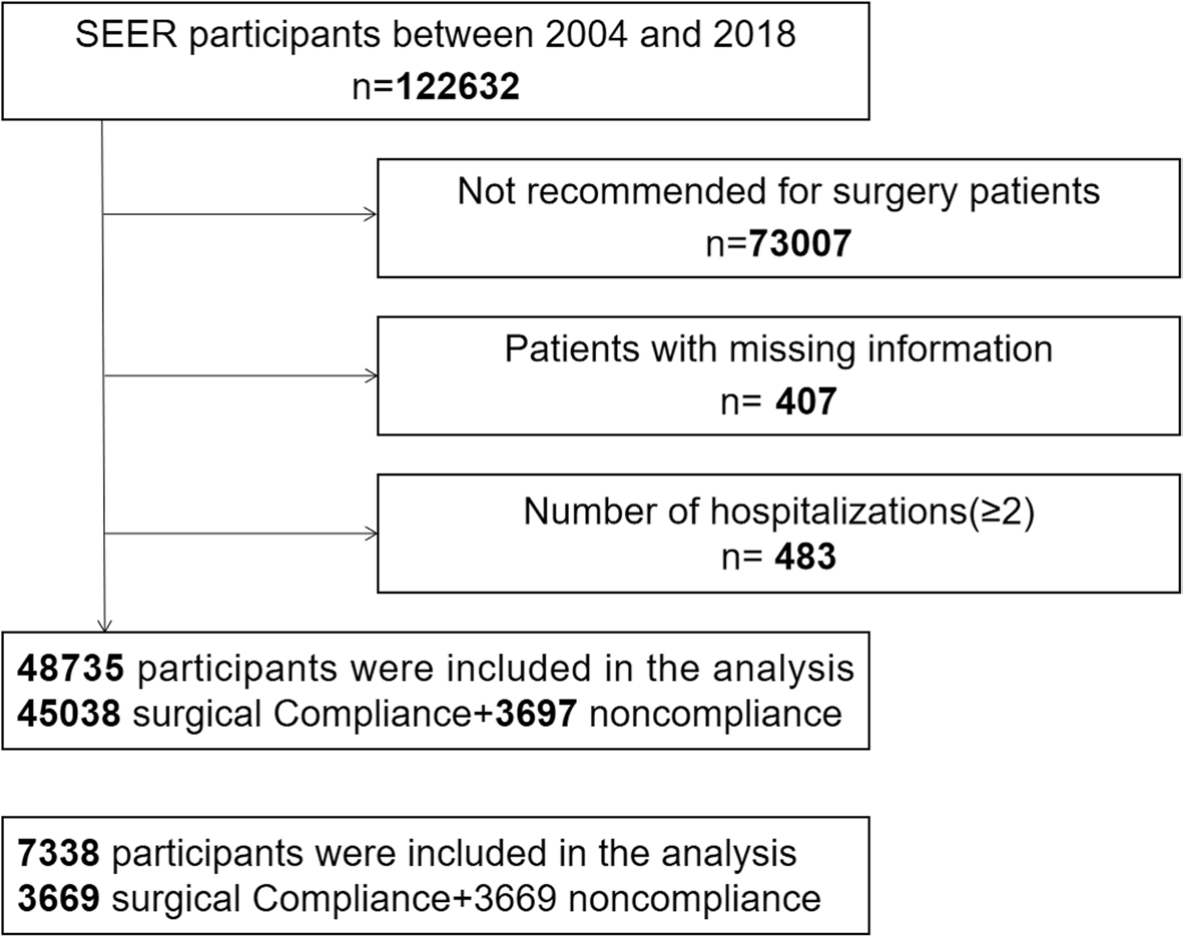
Schematic overview of patient identification

Exclusion criteria were applied (Fig. 1): (1) Patients who did not receive surgery (*n* = 73,007); (2) patients with unknown income and missing basic information were excluded because the importance of socioeconomic status in treatment compliance could not be analyzed (*n* = 407); (3) patients with two or more primary sites were excluded because the condition of each site could not be analyzed separately (*n* = 483). Ultimately, 48,735 eligible patients with diagnosed meningioma were retained for the study.

### Study variables

We extracted the following variables from the SEER database: sex, age, race, tumor behavior recodes, tumor size, surgical compliance, survival, family income, and residential setting. For ease of description, patients who accepted surgery were termed the surgical compliance group; those who were recommended for surgery but had no surgical operation were classified into the surgical noncompliance group. The primary outcome of interest was overall survival (OS).

X-tile software is a powerful tool commonly used in medical research to determine optimal cut-off points for continuous variables, such as age and tumor size, to categorize them into meaningful groups. The robustness of the approach lies in its ability to objectively determine the optimal cut-off points based on the statistical characteristics of the dataset, rather than relying on arbitrary or subjective categorizations. This approach enhances the statistical power and clinical relevance of the findings, allowing for more insightful interpretation and potentially guiding treatment decisions. In this study, patients were divided into three age groups according to the X-tile software: < 62 years, 62–77 years, and > 77 years. Similarly, they were categorized based on tumor size as those < 20 mm, 20–62 mm, > 62 mm, and unknown.

### Statistical analysis

The chi-square test was used to analyze categorical variables of demographic and clinical variables between the surgical compliance group and the surgical noncompliance group, including age, race, sex, tumor behavior recodes, tumor size, family income, and residential setting. Kaplan–Meier curves were employed to plot the OS of surgical compliance in different groups. Univariate and multivariate Cox regression models identified hazard ratios (HR) and 95% confidence intervals (CI) for independent prognostic factors associated with OS, including age, sex, race, tumor behavior recodes, tumor size, family income, residential setting, and surgery status. Univariate and multivariate logistic regression analyses were used to analyze surgical compliance risk factors, which were age, sex, race, tumor behavior recodes, tumor size, family income, and residential setting. Independent risk factors for surgical compliance were used to construct diagnostic nomograms, and ROC curves were used to assess their accuracy. Propensity score matching (PSM) (1:1) analysis was used to adjust for differences between patients with poor and good surgical compliance, adjusting for confounding variables, including variables such as age, sex, race, tumor behavior recodes, tumor size, family income, and residential setting. Once the propensity scores are estimated, matching is performed by pairing individuals from the treatment and control groups who have similar or close propensity scores. Through this matching process, individuals who have similar propensities for treatment are selected for comparison, reducing the imbalance between treatment groups and minimizing the potential effects of confounding variables. We analyzed the data using the MatchIt package in R software with a caliper value of 0.1 [22]. The effect was assessed using standardized mean deviation (SMD), and statistical significance was set at a *p*-value of 0.05. Statistical analysis was performed using SPSS version 27.0 (IBM SPSS Statistical Software, USA) and R software (version 4.2.3).

## Results

### Data sources

Our cohort for this study consisted of 122,632 patients with meningioma, of whom 48,735 were recommended for surgical treatment and 73,007 for non-surgical treatment. Among patients who suggested accepting surgical treatment, 45,038 (92.40%) followed and underwent surgery, while 3697 (7.60%) refused. Table 1 shows the clinical baseline characteristics of meningioma patients in the surgical compliance and noncompliance groups. The chi-square test results indicated significant differences in surgical compliance based on patient age, sex, race, tumor behavior recodes, tumor size, family income, and residential setting (all *p* < 0.001). Older patients (> 77 years), residents of rural areas, and those with incomes <$50,000 had a higher tendency to refuse surgical treatment. In contrast, younger patients (< 62 years), those living in urban areas, and high income groups (>$70,000) are more inclined to accept surgery.

**Table 1:** Baseline demographic and clinical characteristics of meningioma patients

Characteristics	Total	Surgical Compliance	Surgical Noncompliance	*p* value
	(NO.%)	(NO.%)	(NO.%)	
Total	48,735(100.00%)	45,038(92.40%)	3697(7.60%)	
Age, years				< 0.001
<62	27,183(55.80%)	25,888(57.50%)	1295(35.00%)	
62–77	16,598(34.10%)	15,323(34.00%)	1275(34.50%)	
>77	4954(10.20%)	3827(8.50%)	1127(30.50%)	
Race				< 0.001
White	37,623(77.20%)	34,840(77.40%)	2783(75.30%)	
Black	5813(11.90%)	5326(11.80%)	487(13.20%)	
Others	5299(10.90%)	4872(10.80%)	427(11.50%)	
Sex				< 0.001
Male	14,497(29.70%)	13,508(30.00%)	989(26.80%)	
Female	34,238(70.30%)	31,530(70.00%)	2708(73.2%)	
Behavior recode				< 0.001
Benign	42,948(88.10%)	39,370(87.40%)	3578(87.40%)	
Borderline malignancy	4705(9.70%)	4648(10.30%)	57(1.50%)	
Malignant	1082(2.20%)	1020(2.30%)	62(1.70%)	
Tumor size				< 0.001
<20 mm	4949(10.20%)	4602(10.20%)	347(9.40%)	
20-62 mm	22,951(47.10%)	22,559(50.10%)	392(10.6%)	
> 62 mm	3131(6.40%)	3091(6.90%)	40(1.10%)	
unknown	17,704(36.30%)	14,786(32.80%)	2918(78.90%)	
Income				< 0.001
< $50,000	7031(14.40%)	6213(13.80%)	818(22.10%)	
$50,000 - $59,999	7079(14.50%)	6528(14.50%)	551(14.90%)	
$60,000 - $70,000	15,316(31.40%)	14,347(31.90%)	969(26.20%)	
> $70,000	19,309(39.60%)	17,950(39.90%)	1359(36.80%)	
Rural/Urban				< 0.001
Rural	5308(10.90%)	4627(10.30%)	681(18.40%)	
Urban	43,427(89.1%)	40,411(89.7%)	3016(81.6%)	

SEER, Surveillance, Epidemiology, and End Results; Percentages may not total 100 because of rounding

### Prognostic factors for OS of meningioma patients

**Fig. 2:**
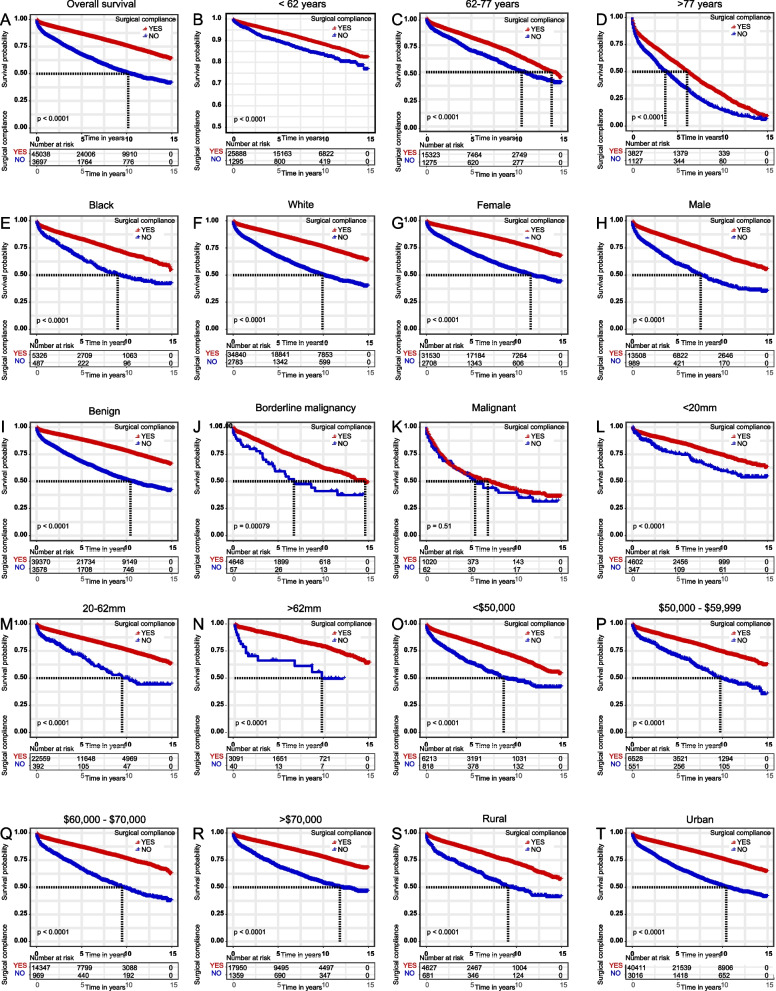
Kaplan–Meier survival curves based on surgical compliance in patients with meningioma. (**A**) OS curves of meningioma patients aged < 62 years, (**B**) 62–77 years, (**C**) and > 77 years. OS curves of meningioma patients segregated by race: (**D**) OS curves in blacks and (**E**) whites. OS curves of meningioma patients segregated by sex: (**F**) OS curves of female and (**G**) male patients. OS curves of meningioma patients based on tumor behavior recode: (**H**) OS curves for benign tumors, (**I**) borderline malignancies, (**J**) and malignant tumors. OS curves of meningioma patients based on tumor size: (**K**) OS curves for those measuring < 20 mm, (**L**) 20–62 mm, (**M**) > 62 mm. OS curves of meningioma patients segregated by household income: (**N**) OS curves for those with a household income of <$50,000, (**O**) $50,000–$59,999, (**P**) $60,000–$70,000, and (**Q**) > $70,000. OS curves of meningioma patients residing in rural (**R**) and urban areas (**S**). OS, overall survival

Kaplan-Meier survival curves were used to assess the effect of surgical compliance on survival (Fig. 2). Our study showed that surgical compliance was significantly associated with OS (*p* < 0.001). Patients with exemplary surgical commitment had significantly better survival than those with poor adherence, and this trend was consistent across all subgroups.

**Fig. 3:**
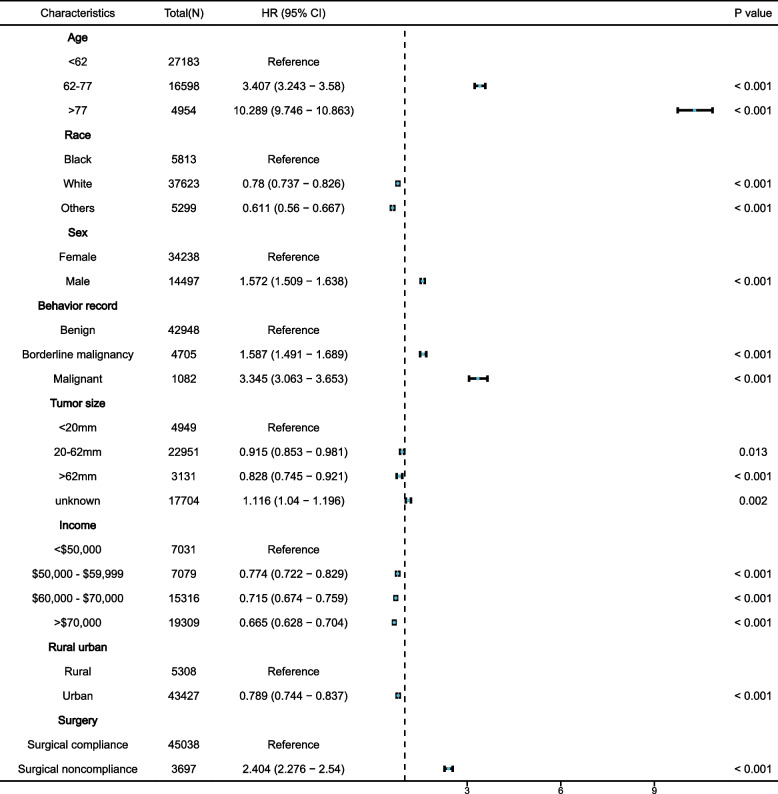
Forest plot of the OS analysis adjusted for age at diagnosis, sex, race, tumor behavior recode, tumor size, income, residential setting, and surgical compliance. The blue dots on the line represent the hazard ratio, and the line represents the 95% confidence interval. Median household income is categorized into equally proportioned quartiles. OS, overall survival

Factors associated with OS in patients with meningioma were analyzed using univariate and multivariate Cox regression analyses. Factors including patient age, sex, race, tumor behavior recodes, tumor size, family income, residential setting, and surgical compliance affected the OS (Table 2). Univariate Cox regression revealed an HR = 2.404 (95% CI 2.276–2.540, *p* < 0.001) for surgical noncompliance compared to surgical compliance. Multivariate Cox regression showed an HR of 1.564 (95% CI 1.471–1.663, *p* < 0.001) for surgical noncompliance, indicating that surgical compliance is an independent prognostic factor for patients with meningioma (Fig. 3).

**Table 2:** Univariate and multivariate analysis of OS rates before PSM

Characteristic	Univariate analysis		Multivariate analysis	
	HR (Hazard Ratio) (95% CI)	*p value*	HR (Hazard Ratio) (95% CI)	*p* value
Age, years				
<62	Reference		Reference	
62–77	3.407 (3.243—3.58)	< 0.001	3.359 (3.196—3.53)	< 0.001
>77	10.289 (9.746—10.863)	< 0.001	9.642 (9.117—10.197)	< 0.001
Race				
Black	Reference		Reference	
White	0.78 (0.737—0.826)	< 0.001	0.694 (0.655—0.736)	< 0.001
Others	0.611 (0.56—0.667)	< 0.001	0.612 (0.559—0.669)	< 0.001
Sex				
Female	Reference		Reference	
Male	1.572 (1.509—1.638)	< 0.001	1.484 (1.424—1.547)	< 0.001
Behavior recode				
Benign	Reference		Reference	
Borderline malignancy	1.587 (1.491—1.689)	< 0.001	1.532 (1.438—1.632)	< 0.001
Malignant	3.345 (3.063—3.653)	< 0.001	2.975 (2.723—3.251)	< 0.001
Tumor size				
<20 mm	Reference		Reference	
20-62 mm	0.915 (0.853—0.981)	0.013	1.018 (0.949—1.092)	< 0.625
> 62 mm	0.828 (0.745—0.921)	< 0.001	0.93 (0.836—1.034)	< 0.181
unknown	1.116 (1.04—1.196)	0.002	1.006 (0.937—1.08)	< 0.868
Income				
< $50,000	Reference		Reference	
$50,000 - $59,999	0.774 (0.722—0.829)	< 0.001	0.87 (0.809—0.937)	< 0.001
$60,000 - $70,000	0.715 (0.674—0.759)	< 0.001	0.794 (0.742—0.851)	< 0.001
> $70,000	0.665 (0.628—0.704)	< 0.001	0.729 (0.681—0.781)	< 0.001
Rural/Urban				
Rural	Reference		Reference	
Urban	0.789 (0.744—0.837)	< 0.001	1.02 (0.951—1.094)	0.582
Surgical				
Surgical compliance	Reference		Reference	
Surgical noncompliance	2.404 (2.276—2.54)	< 0.001	1.564 (1.471—1.663)	< 0.001

PSM, propensity score matching; OS, Overall survival; ^a^Model was adjusted by age, sex, behavior recode, tumor size, income, rural/urban and surgical compliance and income

### Nomogram for OS in meningioma patients

**Fig. 4:**
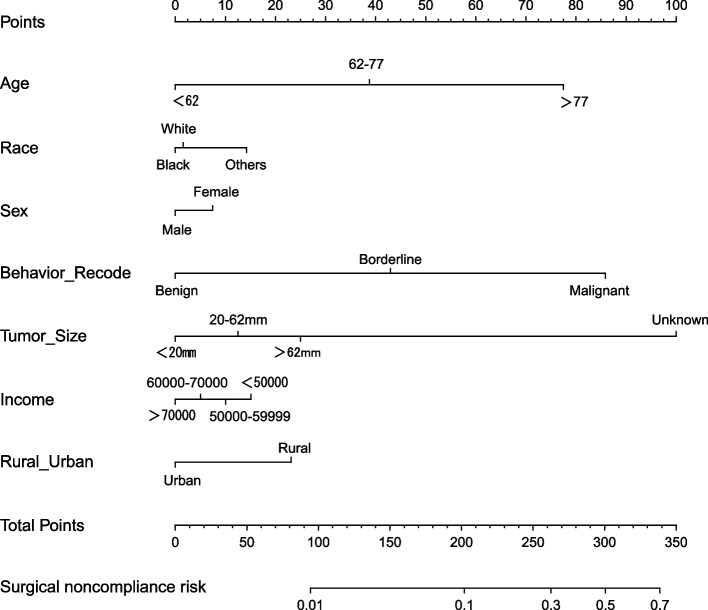
Nomogram for predicting surgical compliance in meningioma patients based on the training set. OS, overall survival

**Fig. 5:**
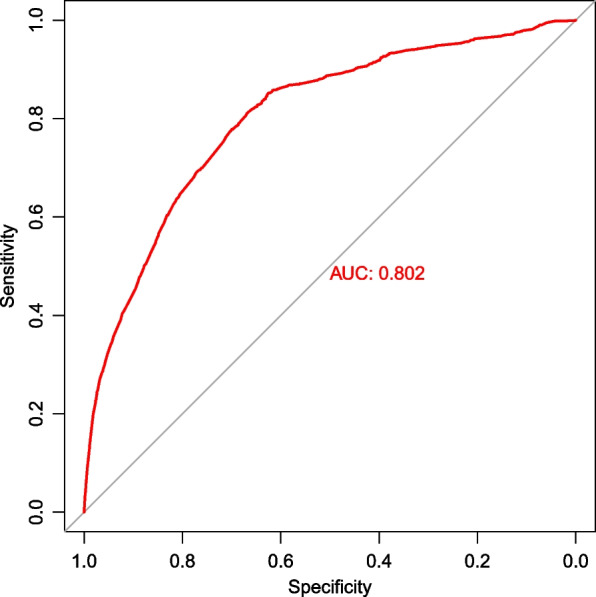
Receiver operating characteristic (ROC) curve analysis of the nomogram for surgical compliance

After the above analysis, we developed a prediction model using seven variables: age, sex, race, behavior recodes, tumor size, income, and residential setting (*p* < 0.05). A nomogram was constructed using these seven predictors to predict the risk ratio for surgical noncompliance in patients with meningioma (Fig. 4). Furthermore, the ROC curve for surgical compliance was plotted, and the area under the curve (AUC) value was 0.802 (95% CI 0.795–0.810, Fig. 5), indicating the reliability of the diagnostic nomogram.

### Surgical compliance factors

Univariate and multivariate logistic regression analyses explored factors related to surgical compliance in meningioma patients (Table 3). In multivariate logistic regression models, borderline malignancy (OR 0.133; 95% CI 0.102–0.174, *p* < 0.001) and older age (> 77 years: OR 6.231; 95% CI 5.666–6.852, *p* < 0.001) had the most significant impact on surgical compliance. Patients who were white race (odds ratio [OR] 0.825; 95% CI 0.739–0.922, *p* < 0.001), male (OR 0.862; 95% CI 0.794–0.935, *p* < 0.001), malignant tumors (OR 0.606; 95% CI 0.461–0.797, *p* < 0.001), larger tumor size (> 62 mm: OR 0.174; 95% CI 0.125–0.243, *p* < 0.001), higher income (> 70,000: OR 0.750; 95% CI 0.664–0.848, *p* < 0.001), and those residing in urban areas (OR 0.581; 95% CI 0.515–0.656, *p* < 0.001) were associated with a higher likelihood of undergoing surgery. However, older age (62–77 years: OR 1.644; 95% CI 1.513–1.787, *p* < 0.001) and other races (OR 1.200; 95% CI 1.032–1.395, *p* = 0.018) were related to poor surgical compliance.

**Table 3:** Univariate and multivariate analysis of variables related to surgical compliance before PSM

Characteristic	Univariate analysis		Multivariate analysis	
	OR (Odd Ratio) (95% CI)	*p value*	OR (Odd Ratio) (95% CI)	*p value*
Age, years				
<62	Reference	< 0.001	Reference	
62–77	1.663 (1.536—1.802)	< 0.001	1.644 (1.513—1.787)	< 0.001
>77	5.887 (5.398—6.421)	< 0.001	6.231 (5.666—6.852)	< 0.001
Race				
Black	Reference	0.012	Reference	
White	0.874 (0.79—0.966)	0.008	0.825 (0.739—0.922)	< 0.001
Others	0.959 (0.837—1.098)	0.540	1.2 (1.032—1.395)	0.018
Sex				
Female	Reference	< 0.001	Reference	
Male	0.852 (0.79—0.919)	< 0.001	0.862 (0.794—0.935)	< 0.001
Behavior recode				
Benign	Reference	< 0.001	Reference	
Borderline malignancy	0.135 (0.104—0.176)	< 0.001	0.133 (0.102—0.174)	< 0.001
Malignant	0.669 (0.516—0.866)	0.002	0.606 (0.461—0.797)	< 0.001
Tumor size				
<20 mm	Reference	< 0.001	Reference	
20-62 mm	0.23 (0.199—0.267)	< 0.001	0.238 (0.204—0.276)	< 0.001
> 62 mm	0.172 (0.123—0.239)	< 0.001	0.174 (0.125—0.243)	< 0.001
unknown	2.617 (2.33—2.94)	< 0.001	2.681 (2.377—3.023)	< 0.001
Income				
< $50,000	Reference	< 0.001	Reference	
$50,000 - $59,999	0.641 (0.572—0.718)	< 0.001	0.84 (0.737—0.958)	0.009
$60,000 - $70,000	0.513 (0.465—0.566)	< 0.001	0.766 (0.676—0.869)	< 0.001
> $70,000	0.575 (0.525—0.63)	< 0.001	0.75 (0.664—0.848)	< 0.001
Rural/Urban				
Rural	Reference	< 0.001	Reference	
Urban	0.507 (0.464—0.554)	< 0.001	0.581 (0.515—0.656)	< 0.001

PSM, propensity score matching; ^a^Model was adjusted by age, sex, behavior recode, tumor size, income, rural/urban and surgical compliance and income

### OS in meningioma patients and subgroup analysis after PSM

**Fig. 6:**
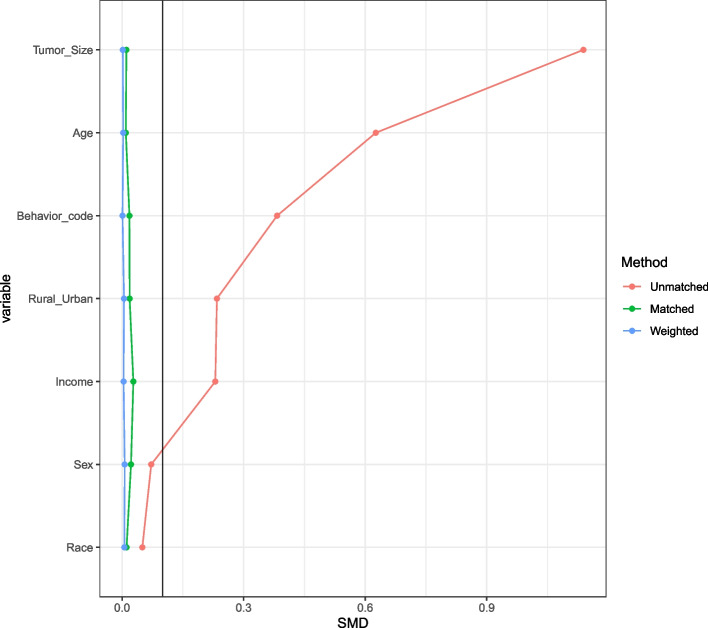
A 1:1 PSM was used to adjust for differences between the surgical compliance and noncompliance groups. PSM, propensity score matching

**Fig. 7:**
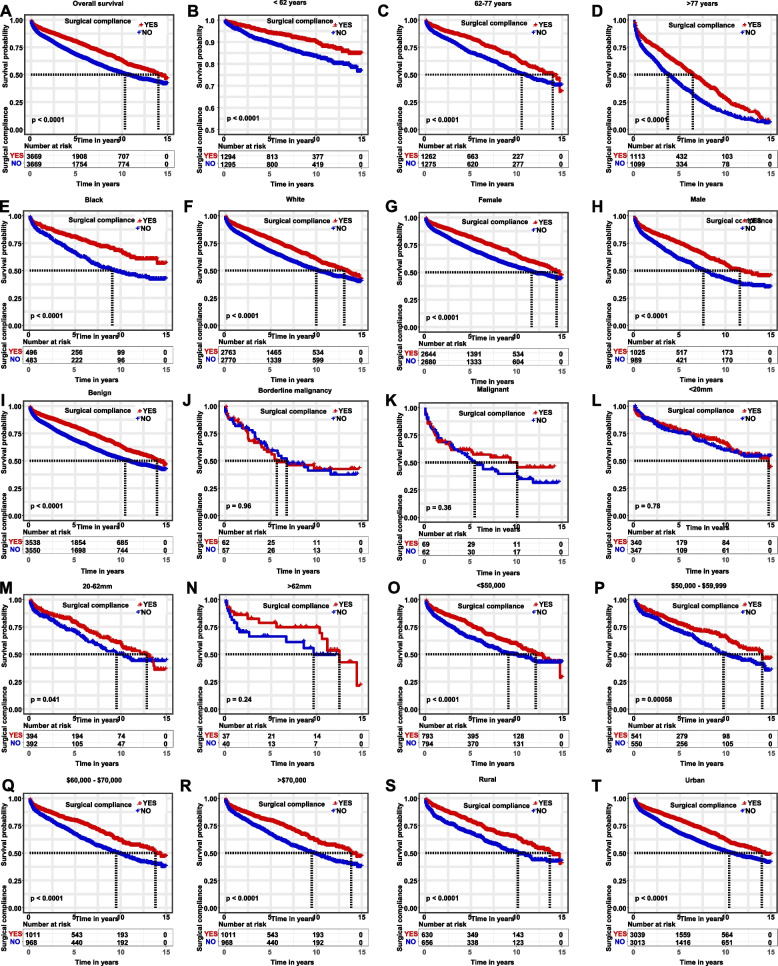
Kaplan–Meier survival curves based on surgical compliance in patients with Meningioma. OS curves for meningioma patients (**A**) < 62 years, (**B**) 62–77 years, and (**C**) > 77 years. OS curves of meningioma patients of different races: (**D**) OS in blacks and (**E**) whites. OS curves of meningioma patients segregated by sex: (**F**) OS in female and (**G**) male patients. OS curves of meningioma patients based on tumor behavior recode: (**H**) OS in benign tumors, (**I**) borderline malignancies, (**J**) and malignant tumors. OS curves of meningioma patients based on tumor size: (**K**) OS curves for those measuring < 20 mm, (**L**) 20–62 mm, and (**M**) > 62 mm. OS curves of meningioma patients based on household income: (**N**) OS curves of those with household income <$50,000, (O) $50,000–$59,999, (P) $60,000–$70,000, and (Q) > $70,000. OS curves of meningioma patients residing in (R) rural and (S) urban areas

After performing a 1:1 PSM to adjust for differences between the number of groups with good and poor surgical compliance (Fig. 6), 7338 patients remained in the analysis with equal numbers in excellent and poor surgical compliance groups. After performing PSM and adjusting for confounding factors, the updated Kaplan–Meier curves persistently showed a significant relationship between surgical compliance and OS meningioma patients (*p* < 0.001). Meningioma patients with good surgical compliance will survive longer (Fig. 7).

Multifactorial Cox regression analysis, after PSM, suggested age, sex, race, tumor behavior recode (benign or malignant), and higher household income (>$70,000) were strongly associated with patient survival. Subgroup analysis based on these variables showed that, across each subgroup (Table 4), meningioma patients with good surgical compliance consistently had higher survival rates than those with poor adherence.

**Table 4:** Univariate and multivariate analysis of OS rates after PSM

Characteristic	Univariate analysis		Multivariate analysis	
	HR (Hazard Ratio) (95% CI)	*p value*	HR (Hazard Ratio) (95% CI)	*p value*
Age, years				
<62	Reference	< 0.001	Reference	< 0.001
62–77	3.638 (3.18—4.162)	< 0.001	3.576 (3.124—4.093)	< 0.001
>77	9.807 (8.624—11.151)	< 0.001	10.165 (8.931—11.569)	< 0.001
Race				
Black	Reference	< 0.001	Reference	< 0.001
White	1.054 (0.938—1.183)	0.376	0.84 (0.745—0.946)	0.004
Others	0.688 (0.574—0.823)	< 0.001	0.681 (0.567—0.818)	< 0.001
Sex				
Female	Reference		Reference	
Male	1.386 (1.275—1.506)	< 0.001	1.446 (1.33—1.572)	< 0.001
Behavior recode				
Benign	Reference	< 0.001	Reference	< 0.001
Borderline malignancy	1.523 (1.173—1.976)	0.002	1.286 (0.99—1.67)	0.059
Malignant	1.661 (1.306—2.114)	< 0.001	1.641 (1.289—2.09)	< 0.001
Tumor size				
<20 mm	Reference		Reference	0.224
20-62 mm	1.215 (1.002—1.473)	< 0.013	1.204 (0.992—1.461)	0.06
> 62 mm	1.323 (0.894—1.956)	< 0.001	1.277 (0.862—1.89)	0.222
unknown	1.174 (1.011—1.364)	0.002	1.152 (0.99—1.342)	0.067
Income				
< $50,000	Reference	< 0.001	Reference	< 0.001
$50,000 - $59,999	0.888 (0.78—1.011)	0.073	0.883 (0.769—1.015)	0.08
$60,000 - $70,000	0.921 (0.825—1.027)	0.139	0.907 (0.797—1.033)	0.142
> $70,000	0.804 (0.724—0.893)	< 0.001	0.775 (0.682—0.882)	< 0.001
Rural/Urban				
Rural	Reference		Reference	
Urban	0.968 (0.875—1.07)	0.525	1.062 (0.936—1.204)	0.352
Surgical				
Surgical Compliance	Reference		Reference	
Surgical Noncompliance	1.469 (1.357—1.59)	< 0.001	1.56 (1.441—1.689)	< 0.001

PSM, propensity score matching; OS, Overall survival; ^a^Model was adjusted by age, sex, behavior recode, tumor size, income, rura/urban and surgical compliance and income

Univariate and multivariate logistic regression analyses after PSM showed no significant relationship between calibrated surgical compliance and study variables (*p* > 0.05) (Table 5).

**Table 5:** Univariate and multivariate analysis of variables related to surgical compliance after PSM

Characteristic	Univariate analysis		Multivariate analysis	
	OR (Odd Ratio) (95% CI)	*p value*	OR (Odd Ratio) (95% CI)	*p value*
Age, years				
<62	Reference	0.925	Reference	0.917
62–77	1.01 (0.905—1.126)	0.865	1.013 (0.907—1.131)	0.817
>77	0.987 (0.881—1.105)	0.817	0.989 (0.882—1.109)	0.848
Race				
Black	Reference	0.894	Reference	0.985
White	1.03 (0.899—1.179)	0.675	1.012 (0.881—1.163)	0.865
Others	1.042 (0.866—1.254)	0.664	1.014 (0.838—1.227)	0.887
Sex				
Female	Reference		Reference	
Male	0.952 (0.859—1.055)	0.346	0.951 (0.858—1.054)	0.336
Behavior recode				
Benign	Reference	0.739	Reference	0.75
Borderline malignancy	0.916 (0.638—1.317)	0.636	0.919 (0.639—1.323)	0.651
Malignant	0.896 (0.634—1.266)	0.532	0.896 (0.634—1.268)	0.537
Tumor size				
<20 mm	Reference	0.977	Reference	0.983
20-62 mm	0.975 (0.794—1.196)	0.807	0.981 (0.799—1.205)	0.855
> 62 mm	1.059 (0.661—1.697)	0.811	1.064 (0.663—1.707)	0.796
unknown	0.977 (0.834—1.145)	0.774	0.982 (0.837—1.152)	0.824
Income				
< $50,000	Reference	0.702	Reference	0.655
$50,000 - $59,999	1.015 (0.87—1.185)	0.846	1.043 (0.883—1.232)	0.618
$60,000 - $70,000	0.956 (0.838—1.091)	0.507	0.994 (0.851—1.161)	0.935
> $70,000	1.024 (0.904—1.159)	0.712	1.066 (0.914—1.242)	0.415
Rural/Urban				
Rural	Reference		Reference	
Urban	0.952 (0.844—1.074)	0.425	0.932 (0.802—1.084)	0.361

PSM, propensity score matching; ^a^Model was adjusted by age, sex, behavior recode, tumor size, income, rural/urban and surgical compliance and income

## Discussion

Our study utilized the SEER database to investigate the effect of surgical compliance on patients with meningioma and found that surgical compliance was an independent prognostic factor for OS in these patients. The survival analysis revealed that meningioma patients with good surgical compliance will survive longer. Worryingly, there is a trend that more patients are opting for conservative management, warranting attention to this subgroup’s disease status and prognosis.

Prior research has demonstrated the crucial role of surgical compliance in the survival of cancer patients. Wang et al. found that surgical compliance was an independent prognostic factor for survival in patients with stage T1–2 non-small cell lung cancer [23]. Good surgical compliance positively correlated with higher survival rates and longer median survival time. Similarly, Liu et al. demonstrated poor surgical compliance in gastric cancer patients exhibited lower survival rates than non-surgically treated patients [24]. Adesunkanmi et al. investigated 212 Nigerian patients with breast cancer, revealing a strong association between surgical compliance and patient prognosis, with most patients dying or being lost after one year [25]. Adham et al. found that survival outcomes were correlated with treatment compliance in Indonesian nasopharyngeal cancer patients [26].

Our logistic regression analysis indicated a strong association between patient age stratification and surgical compliance. Mortality increased significantly with age. We further stratified by age and found that surgical compliance remained an independent risk factor for OS. Meanwhile, numerous studies have established age as an independent risk factor for tumor survival in various cancers, including kidney, cervical, breast, pancreatic, and stomach [27–29]. Steinberger et al. revealed that old age was an independent predictor of morbidity and mortality in patients who underwent craniotomy for meningioma [13]. According to our logistic regression analysis, older age was associated with poorer surgical compliance, especially among those > 77 years of age, who were more likely to refuse surgery by 4–5 times compared to other patients. This trend could be attributed to the poorer physical condition of older patients, the prevalence of various chronic conditions such as hypertension, diabetes, and heart disease, higher surgical risks, and perioperative complications. These factors could result in older patients opting for more conservative and unfavorable treatment approaches, leading to poorer adherence to surgical recommendations [12]. Moreover, surgical treatment of meningioma in older patients is associated with higher complication rates [10]. Ostrom et al. suggested that meningioma are more likely to affect women than men [30]. One study comparing the outcomes of young and elderly patients with meningioma found no statistically significant differences in postoperative mortality, complications, and duration of hospital stay between the two groups. Also, there was no significant difference in good recovery between the two groups in the long postoperative recovery [11]. Therefore, performing surgery in elderly patients seems necessary to achieve good results in patients with meningioma. While the study elicited that women have worse surgical compliance than men, this difference could be attributed to high family pressure and their intention to avoid a financial burden on their families.

Patients who were white race were associated with a higher likelihood of undergoing surgery, so the disparity in the research group might contribute to this discrepancy. We explored the association between family income and patient compliance with surgery in patients with meningioma. Income level was identified as a significant predictor of survival time in these patients. The growing attention to the impact of socioeconomic status on cancer prognosis holds substantial importance, particularly in the context of CNS tumors, which significantly influence patient outcomes due to the unique characteristics of the disease, the high costs incurred, and the complex nature of diagnostic and treatment procedures [31]. Higher household incomes are associated with better socioeconomic status and a higher likelihood of patients opting for surgical treatment [22]. This finding is consistent with the current study analysis, where patients with higher household incomes were more inclined to receive surgical treatment. The financial background of patients plays an essential factor in their treatment choices and surgical compliance.

In addition to these demographic factors, clinical factors were also investigated through logistic regression; tumor malignancy was strongly associated with surgical compliance. Patients with benign tumors showed poorer adherence, possibly due to underestimating the seriousness of the disease. Conversely, surgical compliance is affected by tumor size. With a progressive increase in tumor size, patients might experience more pronounced symptoms of brain compression, leading to headaches, nausea, vomiting, increased intracranial pressure, and even epilepsy. Patients are more willing to accept the surgical treatment to relieve symptoms at this stage.

The results from the Cox regression analysis indicated that surgical compliance was associated with multiple variables related to survival. After performing 1:1 PSM, Kaplan–Meier curve analysis showed that patients with poor surgical compliance had worse median survival prognoses than those with good surgical compliance, consistent with previous findings. These results suggest that differences in treatment compliance may be an essential factor affecting the prognosis of patients with meningioma. Treatment is based on good treatment compliance for patients, alone or in combination. Poor compliance with surgery may indicate poor compliance with other treatment options, ultimately leading to adverse outcomes.

Nomograms are highly effective in predicting survival in patients [32]. As previously reported, our study found that surgical compliance had a non-negligible impact on the prognosis of meningioma patients, so we developed a nomogram to evaluate its ability to predict the risk ratio for surgical noncompliance in patients with meningioma. Therefore, age, sex, race, behavior recodes, tumor size, income, and residential setting can be used to predict the prognosis of meningioma patients. For patients with older age, black and other races, females, advanced-stage tumors, larger tumor size, lower household income, and rural residence, clinicians should fully assess the patients’ condition and develop strategies that will help develop improved compliance and patients’ outcomes.

While this is the first study to focus on risk factors of surgical compliance and its impact on survival outcomes in meningioma patients, our study has some limitations that should be acknowledged. Firstly, the SEER database lacks important confounders associated with surgical compliance, such as detailed information about the patient’s medical condition and comorbidities. Secondly, our data lacked comprehensive information on whether patients received radiation, chemotherapy, or targeted therapy. Thirdly, the large sample size of 17,704 cases with missing and blank data in the categorization of tumor volume size could have potentially introduced bias, affecting the reliability of the study’s results. The SEER database is based on US population characteristics, which limits the generalizability of the study findings to populations in other countries with different healthcare systems and socioeconomic backgrounds. Finally, we did not choose to investigate tumor-specific death as an outcome measure, which could provide valuable insights into the impact of surgical compliance on disease-specific survival in patients with meningioma. The relatively low proportion of borderline and malignant meningioma cases (5787 out of 122,632) might introduce bias in the analysis. Therefore, employing data from an extended period, encompassing changes in treatment regimens, tumor incidence, and OS trends, could introduce confounding factors that influence the final analysis. Meanwhile, the SEER database does not use the commonly used Simpson grading method for surgical resection, limiting our ability to use relevant data. Therefore, more prospective studies are needed in the future to compare the nature of tumors in patients in the compliance and noncompliance groups, increase the proportion of patients analyzed for borderline and malignant meningiomas, specific information on treatment regimens, surgical grading methods, tumor-specific death, and differences in other underlying medical conditions.

## Conclusions

The study’s findings suggested surgical compliance as an independent prognostic factor for survival in patients with meningioma according to the retrospective SEER database, which may provide some implications for future research. Better surgical compliance led to improved patient survival, while poorer surgical compliance was associated with poorer OS. The poor surgical compliance for meningioma patients was related to order age, black and other races, female, advanced-stage tumors, larger tumor size, lower household income, and rural residence.

## Data Availability

The dataset analyzed during the current study are available from the corresponding author upon reasonable request (zhouhai3212@163.com).
